# Novel combination therapies for BRAF-mutant melanoma

**DOI:** 10.1186/1479-5876-13-S1-K6

**Published:** 2015-01-15

**Authors:** Grant A McArthur

**Affiliations:** 1 Peter MacCallum Cancer Centre, St Andrews Place, East Melbourne, Victoria, Australia, 3002; 2 University of Melbourne, Parkville, Victoria, Australia

## 

The ability to inhibit the activity of the MAPK pathway in BRAF-mutant melanoma has led to profound improvements in clinical outcomes for patients with advanced BRAFV600-mutant melanoma. Notably the additional of a MEK-inhibitor to a type-I BRAF inhibitor results in greater inhibition of MAPK-signaling resulting in improved partial and complete response rates, and prolongation of progression free and overall survival without increasing toxicity in normal cells. Three phase 3 clinical trials have shown highly consistent clinical outcomes defining the combination of a BRAF and MEK-inhibitor as a new standard of care for advanced BRAFV600-mutant melanoma. However resistance to the combination therapy occurs in the vast majority of patients necessitating the development of further novel strategies to overcome resistance. Intriguingly early genomic analyses of tumor tissue from patients progressing on combined BRAF and MEK-inhibition suggest reactivation of the MAPK-signaling as one mechanism of resistance. One strategy to overcome this is to inhibit MAPK-signaling downstream at key signaling nodes such as the cell cycle regulator CDK4. Our preclinical studies show that adding the CDK4-inhibitor palbociclib to a BRAF inhibitor will prevent the emergence of resistance. An alternative strategy is to combine BRAF-inhibitors with therapies that target other mechanisms of disease control such as the host response or tumor metabolism. It is clear that therapeutic combinations either sequentially or concurrently, will continue to define new standards of care for patients with advanced BRAF-mutant melanoma.

